# The Condition of Dentine in Pulpless Teeth

**Published:** 1892-10

**Authors:** W. C. Barrett

**Affiliations:** Buffalo, N. Y.


ARTICLE II.

### THE CONDITION OF DENTINE IN PULPLESS TEETH.

BY DR. W. C. BARRETT, BUFFALO, N. Y.

Read in the Section of Dental and Oral Surgery, at the Forty-third Annual Meeting of the American Medical Association, held in Detroit, Mich., June, 1892.

I had promised the Secretary of this Section that I would prepare and read a paper at this meeting upon the "Condition of the Dentine in Pulpless Teeth." It was a subject upon which I had bestowed some thought, but with the literature of which I had not made myself thoroughly acquainted. I had read Prof. Miller's work on "The Micro-organisms of the Human Mouth," and his communications to the various journals, notably *The Dental Cosmos*, but not with this special subject in mind. I had not collocated his writings upon this theme, and hence had not collated them. When in pursuance of my plan this had been done, I was astonished at the thorough manner in which the ground had been covered.

Had I examined my subject earlier, I should not have attempted an excursion in this field; but having engaged to present this paper, I will endeavor to redeem my promise, premising at the outset that I can do little save to call attention to what has been done by Prof. Miller in this direction, a work which does not seem to be fully appreciated, and which perhaps will not be until each attempts, like me, specially to study it for himself. In view of his exhaustive experiments, one can but wonder at the seemingly crude theories yet held and taught by some men to whom we naturally turn for illumination. Perhaps I shall do more good by placing Dr. Miller's comprehensive studies before you for discussion, than I could by effort in any other direction, for it must be remembered that his are not mere generalizations, such as mine must necessarily be; they are the results of a long series of conclusive, scientifically conducted, original experiments and observations, and they are not to be overthrown by any inductive reasoning whatever. In view of the overwhelming original testimony which he introduces, no man has the right to dispute his deductions until he is fortified by a like mass of opposing facts, deduced from like carefully conducted experiments or until he can indisputably demonstrate that the conclusions reached by Dr. Miller are not warranted by the results obtained. Mere mental speculation, however plausible, cannot be accepted against thoroughly demonstrated facts.

The dentine, deriving its nutriment from the pulp of the tooth through the dentinal fibrillæ, is separated from all the other tissues of the body, through investment by the cementum and the enamel. The latter tissue is not only comparatively devoid of living matter, but it is not in direct relation with any tissues save the dentine itself. For our present purpose, the enamel may be looked upon as a simple protecting cap, whose office it is to preserve the dentine and pulp from injury.

The cementum is of a different character. It is but slightly differentiated from true bone. It is in relation with a periosteal membrane and it is nourished like bone through a system of lacunal corpuscles, connected by canaliculi. In young teeth it is not infrequently the case that there is even a kind of system of H[illegible]versian canals, traversing the cementum and dentine between the pericementum and pulp, whose office it is to assist in maintaining the vascular supply, and incidentally, perhaps, to afford nutriment to the deeper cementum corpuscles.

The dentine is yet more differentiated, and in its structure has lost the lacunal corpuscles, while the canaliculi are modified into the dentinal fibrillæ. Their office is not materially changed, nor are we to suppose that the contents of the dental tubuli differ very widely from those of the canaliculi of true bone. They form the living portion of the dentine, as the lacunal corpuscles and the canaliculi form that of the bone. The dentinal fibrillæ are in connection with the vascular pulp, and from it derive the nutriment necessary to their continued life. The corpuscles found in the bone and in cementum are absent, and hence there is little that bears a resemblance to that reticulum of true bone which forms its lamellated structure. At the extremities of the dentinal fibrillæ, we have the irregular interglobular spaces which mark the boundary line between dentine and enamel, and which may, perhaps, be considered as modifications of the cementum corpuscles, but they are not intimately connected with the dentinal structure.

The dentinal fibrillæ, depending as they do upon the vascular pulp for nutrition, certainly lose their protoplasmic vitality when separated from it. Let us consider for a moment the condition of the dentine in a tooth, the pulp of which has, without medication, just been forcibly removed. The fibrillæ have been freshly torn from their relations with the pulp, and remain, filling the dentinal tubuli as before their violent separation. Like all protoplasmic material, they are composed of the great quaternary of elements which enter into the composition of all organic elementary structures. But the dentinal fibrillæ probably also contain other elements, such as sulphur and phosphorus, which make it more near the nature of albumen. Indeed, it is probable that if we consider it as clearly albuminous, we shall not do much violence to nature. Carpenter says tnat this substance is the proper pabulum of the animal tissues generally, and so the dentinal fibrillæ, possibly modified somewhat, may be looked upon as albuminous in their nature.

The dentinal tubules, which contain the fibrillæ, are exceedingly minute. For the putrefaction of the contents of the tubuli, it would be essential that putrefactive organisms should enter them. While it is quite possible that these may penetrate the tubuli, it must be to but a limited extent. In the article in *The Dental Cosmos* for May, 1890, upon "The Decomposition of the Contents of the Dental Tubuli," Dr. Miller shows that from their minute size and the very small amount of nutriment for the micro-organisms which they contain, any bacteria which enter them must soon perish from want of pabulum, to say nothing of the fact that they are mostly ærobic, and so could not possibly live in the tubules for any length of time.

But not satisfied with *apriori* reasoning, he commences a rather remarkable series of experiments and observations, from which he incontestibly deduces the conclusion that bacteria do not penetrate the tubuli of undecayed dentine to any marked extent; certainly not enough to cause any necessity for the disinfection of the dentinal substances, in teeth in which there has been no decay within the pulp canals. I need not enlarge upon this. The record is open to the inspection of all, and no one has yet dared to take issue with Dr. Miller upon this point. It must be accepted, that in the dentine of the roots of teeth in which decay has made no progress, there is no infection of dentine that demands any antiseptic treatment, aside from that usual in pulp canals.

What then becomes of the contents of the dentinal tubuli? It is but necessary to consider their character to form a clear estimate of this. The dentinal fibres cannot be considered as pure albumen, for that is only found in connection with other substances in the body. But whatever its exact composition, it presents certain characteristics, one of which is its tendency, when at rest and separated from living tissue, to dissociate the watery portions, and to form a dried residuum which is insoluble. This condition may also be produced by most acids, by the metallic salts, and by creosote. The natural tendency, then, for an albuminoid fibril, would be to assume a mummified condition, most conducive to its indefinite preservation when kept in the state in which it will be found in dentine of a tooth root.

Fibrin is but comparatively slightly differentiated from albumen. It contains the same elements, but it offers certain physical characteristics that distinguish it. Albumen is coagulated by heat, and by certain agents. Fibain coagulates spontaneously when exposed to the air. If, then, the fabrillæ of the dentine approach fibrin in their characteristics, they will undergo the change called coagulation without the intervention of any medicinal agent whatever. It is in this condition that, if sealed up from external influences, they present the very best state for the preservation of the tissue in which they are found. Hence that condition of the dentine is best in which the contents of the tubuli have been coagulated, or separated from the watery elements, and permitted to become a kind of minute mummy without their investing sheaths.

But the investigations of Miller, to which I have already referred, determine another matter. He did not find that the mouths of the dental tubuli, in teeth from which the pulp had been removed, were opened toward the pulp chamber. He had under examination nineteen teeth. In ten of the roots of these he found the canals to be completely or partially lined with a homogeneous structure, containing very few or no tubuli, but occasionally a formation resembling a bone lacuna, and bearing a much more near resemblance to cementum than to dentine. This layer is impenetrable to micro-organisms, and therefore forms a protection to that tissue when the pulp has been removed. We find, then, that the dentine is not only protected by the cementum on its external aspect, but very often by a modification of this tissue on its inner surface.

There is, therefore, very little danger of infection of the contents of the dental tubuli But even if this were possible, I cannot conceive that it would matter much. The amount of infectious matter would be so small, and the chance for the penetration of the gaseous products of decomposition through the dentinal tubuli, past the zone of demarcation between dentine and cementum, and finally though the cementum corpuscles and canaliculi, which are themselves filled with living matter, would be so remote, and its influence upon the pericementum so infinitesimally small, that it seems folly to take it into consideration in the presence of so much more potent factors.

Clinical experience, too, as is so plainly stated by Dr. Miller in the article to which reference has been made, does not sustain the theories of those who insist upon the possibility of infection from dentine. If the contents of the pulp canal be completely removed, the territory made thoroughly aseptic and the space finally filled, there is, so far as I am aware, no record of any trouble from the dentine. Even admitting that there exists within the dentinal tubuli matter which is susceptible to putrefactive changes, it is too small in amount to induce any disturbing condition in the presence of a pulp canal, carefully filled with a material which must seal the exceedingly minute mouths of the tubules wherever they are not already stopped by the modified dentinal layer of which Miller speaks. In the presence of so much more important things, it seems like bad judgment to elevate such a mole-hill into a mountain. It is taking tithes of mint and cumin, and neglecting the mighties matters of the law. To elevate such small matters into prominence in private, has a tendency to induce carelessness in things that need more attention. I doubt whether there ever has been a case of reinfection of the tissues about a tooth that has had its root filled after antiseptic treatment. that cannot be directly traced to some imperfection in the manipulation, or to some infected pockets outside the tooth itself. It is well known that the usual place for the formation of an infected pocket is at or near the apex, and that there may be a penetration of the micro-organisms and the formation of infected pockets for some distance back in the bone. I have myself seen at least three such, extending back from the point of an incisor tooth until the last one was over the second bicuspid, and each of these separate pockets connected with the original one at the apex of the incisor. They were only reached by a long incision through the gum and external alveolus, extending from one to the other until the last one had been reached. If the infection had been from the dentine, it would have manifested itself quite differently.

Undoubtedly there are cases in which the infected spot is at some point in the pericementum between the gingival margin and the apex of the tooth. But this does not by any means prove that it came from infected dentine. Perhaps, after the apex of the tooth, the most usual place for the formation of an abscess is at the bifurcation of the roots of a bicuspid or molar. I believe this to be due to the fact mentioned earlier in this paper, that something analogous to an Haversian canal there penetrates the cementum and dentine, from the pericementum to the pulp. I have demonstrated such many times. Not infrequently an abscess refuses to yield to the usual treatment through the pulp canal, and for the reason that the medicaments fail to reach the infected point. But such instances cannot be said to be produced by infected dentine. There is, in truth, another foramen analogous to that at the apex, and it becomes septic in precisely the same manner as does the latter.

In what I have previously urged, we have presupposed that caries had not commenced within the pulp canal. And yet we are well aware that in cases in which the pulps had been long dead, and there has been extensive decay of the coronal portion of the tooth, there was caries of the dentine, commencing within the pulp chamber and extending down the canal. In instances like this, of course there will be thorough infection of the dentine. It will not, however, be due to decomposition of the contents of the dental tubuli, but to infectious matter, which may find its way into the hollow from external sources. It must be as manifestly improper to fill such a canal without removing the carious portions, as it would be to fill a cavity from the outside without careful excavation. Here will be an instance in which the reaming out of the canal to the depth to which caries had penetrated would be eminently proper practice. It is very seldom that this decay will extend far down the root canal, or be very deep, without such destruction of the crown and cervical portions of the root as to make preservation practically impossible.

As regards the use of coagulating agents in the treatment of root canals, I cannot conceive of any objection to them. If by any it be thought necessary that dentine should be disinfected and made aseptic, this can only be accomplished by agents which have the power of penetration in a marked degree. These are usually coagulants, like chloride of zinc, bichloride of mercury, etc. Upon this subject I can only refer to the experiments and observations reported by Dr. Miller, in an article on "The Relative Rapidity with which Various Antiseptics Penetrate Pulp Tissue," published in *The Dental Cosmos* for May, 1891.—*Dental Pract. and Advertiser.*

